# Application of machine learning and genetic optimization algorithms for modeling and optimizing soybean yield using its component traits

**DOI:** 10.1371/journal.pone.0250665

**Published:** 2021-04-30

**Authors:** Mohsen Yoosefzadeh-Najafabadi, Dan Tulpan, Milad Eskandari

**Affiliations:** 1 Department of Plant Agriculture, University of Guelph, Guelph, Ontario, Canada; 2 Department of Animal Biosciences, University of Guelph, Guelph, Ontario, Canada; South China University of Technology, CHINA

## Abstract

Improving genetic yield potential in major food grade crops such as soybean *(Glycine max* L.) is the most sustainable way to address the growing global food demand and its security concerns. Yield is a complex trait and reliant on various related variables called yield components. In this study, the five most important yield component traits in soybean were measured using a panel of 250 genotypes grown in four environments. These traits were the number of nodes per plant (NP), number of non-reproductive nodes per plant (NRNP), number of reproductive nodes per plant (RNP), number of pods per plant (PP), and the ratio of number of pods to number of nodes per plant (P/N). These data were used for predicting the total soybean seed yield using the Multilayer Perceptron (MLP), Radial Basis Function (RBF), and Random Forest (RF), machine learning (ML) algorithms, individually and collectively through an ensemble method based on bagging strategy (E-B). The RBF algorithm with highest Coefficient of Determination (R^2^) value of 0.81 and the lowest Mean Absolute Errors (MAE) and Root Mean Square Error (RMSE) values of 148.61 kg.ha^-1^, and 185.31 kg.ha^-1^, respectively, was the most accurate algorithm and, therefore, selected as the metaClassifier for the E-B algorithm. Using the E-B algorithm, we were able to increase the prediction accuracy by improving the values of R^2^, MAE, and RMSE by 0.1, 0.24 kg.ha^-1^, and 0.96 kg.ha^-1^, respectively. Furthermore, for the first time in this study, we allied the E-B with the genetic algorithm (GA) to model the optimum values of yield components in an ideotype genotype in which the yield is maximized. The results revealed a better understanding of the relationships between soybean yield and its components, which can be used for selecting parental lines and designing promising crosses for developing cultivars with improved genetic yield potential.

## Introduction

Soybean (*Glycine max* L. Merrill) is the world’s most widely grown leguminous crop and an important oil and protein source for food and feed [1]. Because of its nutritional and pharmaceutical properties, soybean is also considered as an important source of healthy plant-based food products in the human diet. While the global demand for soybean is increasing significantly [2], the current average annual genetic gain for yield seem not to be able to cope with the growing demand [3]. One of the probable main reasons for this low genetic gain is the inefficient selection criteria that are currently used in breeding programs for selecting genotypes with desirable genetic yield potentials [4].

Despite the major advances in molecular technologies and their potential implications in breeding programs, generating reliable phenotypic data and analyzing big datasets have been remained the major bottlenecks [5]. Plant breeding programs, including soybeans, are continuously relying on the evaluation of yield and important agronomic traits for making selections and defining commercial products [6]. If a trait is under controlled by a few major genes, and so with limited environmental effects, designing molecular marker tools would be sufficient for selecting desirable genotypes in a given breeding population [7]. However, for complex traits such as yield, which are highly influenced by the environment and controlled by numerous genes with minor effects, the dissection of traits underlying the yield can be beneficial for selecting genotypes with improved genetic potentials [8]. In general, soybean breeders have made extensive use of the classical phenotypic selection approaches to evaluate and exploit total seed yield as the main selection criterion in their cultivar development programs [8, 9]. However, genetic gains for yield have generally been low and inconsistent across different environments [8]. The general low genetic gains for yield can be to a large extent because of the nature of this trait, in which several secondary traits drive the final production directly or indirectly [3]. Thus, one possible way to increase the yield genetic gains in new cultivars is to improve their yield component traits [5, 7, 8].

Yield formation in plant species is mostly governed by yield component traits [10–12]. Traits such as the number of nodes per plant (NP), number of non-reproductive nodes per plant (NRNP), number of reproductive nodes per plant (RNP), and number of pods per plant (PP) are considered as the major yield components in soybean [13]. Therefore, the increase in yield production can be regulated by selecting the soybeans with greater performance in their yield components [13, 14]. Several studies have been conducted to describe yield improvement in plants via improving yield components [15–18]. For example, positive correlations between soybean yield and the number of pods [19] were reported to be the most significant contributor to yield gain in Northeast China [20]. By considering yield components, soybean breeders can model and predict optimum conditions in which the highest yield production can achieve [21]. However, developing reliable prediction models built upon several yield component traits requires dealing with large datasets that are generated from the evaluation of large breeding populations across multi-environments.

Due to the essential need for advanced skills in computational and mathematical analyses, exploiting large datasets in many public breeding programs is still a bottleneck. Machine Learning (ML) algorithms, as one of the reliable and efficient computational approaches, were successfully implemented in different fields of study, such as traffic crash frequency modeling [22, 23], environmental science [24], engineering [25], and medicine [26]. Previously, Zeng, Huang (22) developed neural network, as one of the ML algorithms for exploring the non-linear relationship between risk factors and crash frequency. They reported the successfulness of using neural network algorithms to eliminate the overfitting and deal with lack-box characteristic [22]. Also, several studies reported the efficiency of ML algorithms in better detection of genomic regions associated with a trait of interest [26–28].

One of the recent agriculture trends is the use of ML algorithms for analyzing big data [29, 30]. Emerging ML algorithms in agriculture have created new opportunities to quantify and understand the intensive data process in agriculture [29, 31]. In a simple form, ML algorithms can be defined as machines with the ability to learn without explicitly programmed [29, 32]. Theoretically, each algorithm is involved in a specific learning process from training data to perform a task of clustering, predicting, and classifying new datasets using the knowledge attained during the learning process [31]. Various ML algorithms have been developed and can be implemented for complex interactions between features [8, 29, 32, 33]. Multilayer Perceptron (MLP), for example, is known as the common neural network algorithm that is widely used in different areas such as plant sciences [30], remote sensing [34], environmental sciences [35], and engineering [36]. Like other neural network algorithms, MLP is built upon many neurons in which each neuron has its own specific weight [37]. In any case that one neuron is insufficient to explain the algorithm, MLP will be useful by providing multi-neurons [38, 39]. Radial Basis Function (RBF) is another ML algorithm commonly used in plant sciences [40–42]. RBF is reported to be successful for predictions wherever relevant features are used [40]. However, its performance for predicting soybean yield from its components is still unknown. Random Forest (RF) is another algorithm that its performance was evaluated in this study. RF has drawn many researchers’ attention because of adequate performances in various fields, including plant science [30, 43], animal science [44, 45], human science [46], and remote sensing [47].

One of the major impediments of using individual ML algorithms is the high probability of overfitting in single predictive algorithms [48]. To overcome this obstacle, the ensemble techniques can be employed [49]. Ensemble techniques are known as the most influential development in the application of ML algorithms [50], in which combined algorithms are exploited to improve prediction accuracies by reducing overfitting rates [48–50]. Three commonly used ensemble algorithms are stacking, boosting, and bagging methods that are used according to the nature of the dataset and the individual ML algorithms that are used [50]. The success of using ensemble techniques was reported in different areas such as plant science [30], engineering [51], and computer sciences [52].

In soybean cultivar development programs, an optimum selection among important yield components can significantly improve the yield genetic gain. Therefore, the implementation of optimization methods in this field is of particular interest. Optimization algorithms for improving important traits are becoming more and more attractive in plant science [40, 53]. Genetic Algorithm (GA) is known as one of the most well-known single objective optimization methods designed and developed by Holland [54], as a searching algorithm based on natural selection. GA searching algorithm is based on Darwin’s notion that more stable organisms across different environments survive better than the others [55].

Although the successful uses of ML ensemble methods have been reported in different agriculture-related fields [30, 48, 53, 56, 57], the potential use of these algorithms to predict soybean yield using yield components remains unknown. Therefore, this study aimed to investigate the potential use of soybean yield components for predicting the final seed yield using individual ML algorithms as well as ensemble learning methods. In addition, linking the best machine learning algorithm with GA for estimating the optimized values of the yield components for maximizing the soybean yield was investigated. The outcomes of this study can pave the way to understand the importance of soybean yield components in determining the total seed yield and implement the proposed pipeline for making the genotypic selection more accurate.

## Material and methods

### Plant material and experimental design

Two hundred and fifty soybean genotypes were grown under field conditions at two locations: Palmyra (42°25’50.1"N 81°45’06.9"W, 195 m above sea level) and Ridgetown (42°27’14.8"N 81°52’48.0"W, 200m above sea level), in Ontario, Canada in 2018–2019. The population used in this study was selected from the core germplasms of soybean breeding programs at the University of Guelph, Ridgetown campus that have been created over 35 years and used for cultivar development and genetic studies. The experiment was conducted using randomized complete block designs (RCBD) with two replications in four environments (two locations × two years), consisting of 2000 phenotypic plots in total. Each plot consisted of five rows, each 4.2 m long and 40 cm spacing between each row. The seeding rates used in this study were 50–57 seeds per m^2^.

### Phenotypic evaluations

Seed yield (ton ha^-1^) of each plot was measured by harvesting three middle rows and adjusting to a 13% moisture level. Soybean yield components, including NP, NRNP, RNP, and PP, were hand-measured by randomly selected ten plants from each phenotypic plot for each genotype. Also, the PP to NP ratio (P/N) for each genotype was calculated using the following equation:

$$P/N=\frac{PP}{NP}$$ (1)

where *PP* indicates the number of pods per plant, and *NP* indicates the total number of nodes per plant.

### Data pre-processing, correlation coefficient, and statistical analyses

All the phenotypic plot-based data were adjusted for spatial variations within the fields using nearest neighbor analysis (NNA) as one of the spatial error control methods to reduce and minimize the possible error in the field data [58, 59]. The yield components were collected for 250 soybean genotypes from 2000 plots across four environments. The average value of each yield component for each genotype was estimated through the best linear unbiased prediction (BLUP) as a mixed model [60]. For BLUP analysis, the environment factor was selected as a fixed effect, and the genotype factor was considered as a random effect. Afterward, all 250 data-points were used for constructing training and testing datasets. In this study, all the yield component traits such as NP, NRNP, RNP, PP, and P/N were considered as input variables for predicting the soybean yield as the output variable. In order to improve the prediction accuracy of input variables, data scaling and centering were applied for the pre-processing and pre-treatment steps [61]. Before performing ML algorithms, the principal component (PC) analysis was applied to identify outliers; however, no outlier was detected. The Pearson coefficient of correlations between seed yield and yield components were estimated using the R software version 3.6.1.

### Data-driven modeling

MLP, RBF, and RF are the most commonly used ML algorithms with distinct functions and abilities that are used for predicting yield in plant crop species [37, 48, 62]. The MLP, as one of the most common feed-forward neural networks, consists of input, hidden, and output layers of interconnected neurons [63]. RBF is another type of neural network that used approximate multivariate functions [64]. RBF has the same functionality as that of MLP but in an effective way for using in more than one dimension [65]. RF is also another commonly used ML algorithm that generates combined trees representing *n* number of independent observations [66]. The final prediction in RF is determined based on the average predictions of all possible independent trees [48]. In order to improve the prediction performance of individual ML algorithm, an ensemble method was proposed based on the bagging strategy (E-B). The steps for the E-B analyses are as follows: (1) applying and training MLP, RBF, and RF, independently; (2) selecting the best ML algorithm, based on the validation set, as the metaClassifier for the E-B; and (3) combining the prediction results in order to improve the prediction accuracy of the soybean yield [67]. All the used parameters in each algorithm were optimized based on the training dataset. The Weka software version 3.9.4 [68] was used to run all the tested ML algorithms and the E-B method for analyzing training and validation sets.

### Optimization process via GA

**Fig 1:**
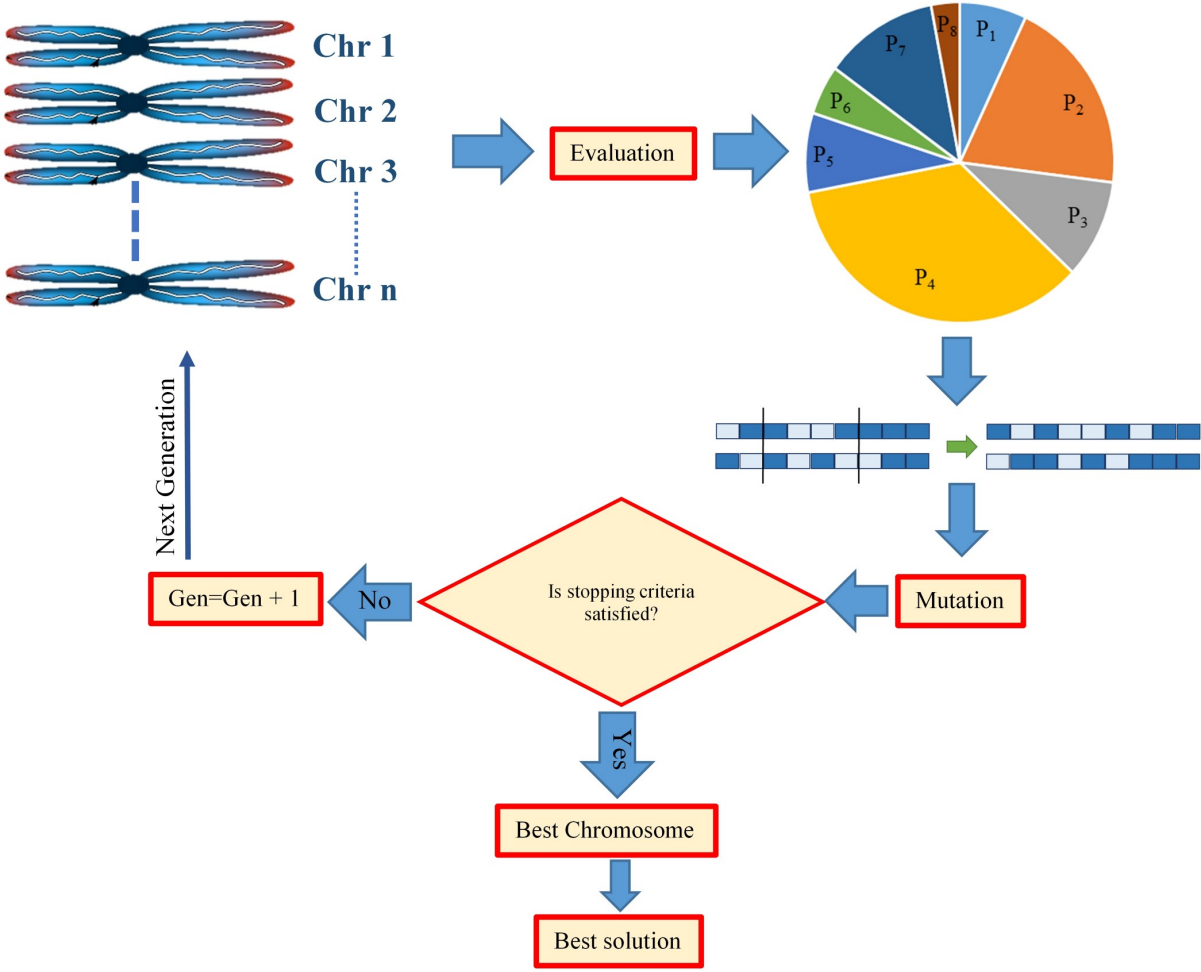
The schematic diagram of the genetic algorithm as the single objective evolutionary optimization algorithm.

For obtimizing the values of NP, PP, RNP, NRNP, and P/N in a theoretical maximized yield ideotype genotype, the best ML algorithm was linked to the genetic algorithm (GA). In order to have the best implement of GA, some parameters such as mutation rate, crossover fraction, and the number of chromosomes should be determined. For that, optimum values of the mentioned parameters are estimated by the trial and error methods. In brief, a chromosome is known as a set of variables that define as a possible solution to the problem. In this study, all the possible combinations of the yield component traits were considered as different chromosomes to form the maximum soybean yield production. All proposed chromosomes are constructed from the initial population, which is the first step in the GA optimization process [53]. Crossover in GA is the process of creating a new generation of chromosomes by mixing the existing chromosomes [69]. In this study, the possible crossover between two chromosomes, each containing a combination pattern of the yield components, was evaluated by using a two-point crossover, which is known as one of the common crossover fractions. Mutation is another parameter in GA, which is used to control the local minima in the population [69]. By using a mutation rate, the possibility of having similar chromosomes will be decreased, and therefore, the possible local minima are decreased [53]. In this study, the mutation and crossover rates as well as the number of chromosomes were set to 0.1, 0.7, and 100, respectively (Fig 1). Also, the Roulette wheel was used for selecting elite populations for crossover to obtain the appropriate fitness. In order to achieve the generation number, the generational practice was iteratively implemented. The lower and upper bounds of the dataset were considered as the constraints in the optimization process (Fig 1).

### Quantification of model performance and error estimations

**Fig 2:**
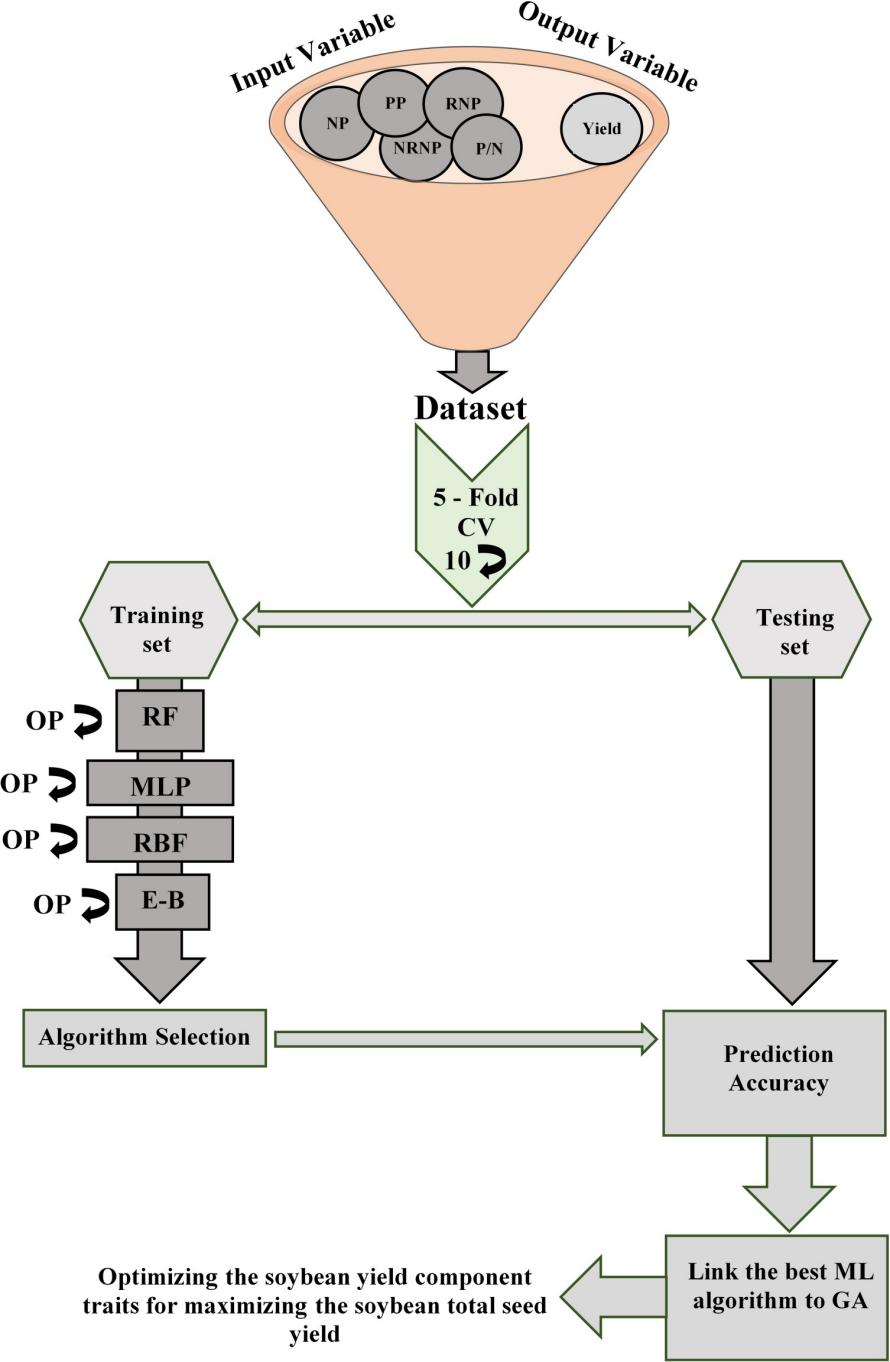
The experimental workflow of algorithm selection and validation for predicting the soybean seed yield. The Number of Nodes per plant (NP), the Number of Non-Reproductive Nodes per Plant (NRNP), the Number of Reproductive Nodes per Plant (RNP), and the Number of Pods per Plant (PP), the ratio of number of Pods to number of Nodes per plant (P/N), Genetic Algorithm (GA).

The original dataset consists of 250 observations was randomly divided into training and validation sets based on the five k-fold cross-validation method [70] with ten repetitions (Fig 2). To quantify the performance of the ML algorithms for predicting soybean seed yield from the yield components, the following statistical measurements between independent reference values (Y) and estimated values (Y′) were applied: The Root Mean Square Error (RMSE, Eq 2) and the Mean Absolute Errors (MAE, Eq 3) of the validation set.

$$RMSE=\sqrt{\frac{\sum {\left(Y^{\prime }-Y\right)}^{2}}{n}}$$ (2)

$$MAE=\frac{\sum_{i=1}^{n}\left|{Y^{\prime }}_{i}-YI\right|}{n}$$ (3)

where *Y′* stands for predicted value, *Y* is the field measured value, and *n* is the number of observations for a given genotype.

The analyses of the *goodness of fit* between observed and the predicted values were performed using the coefficient of determination (R^2^, Eq 4). While we provide their definitions below, more comprehensive descriptions and definitions can be found in [71–73]:

$$R^{2}=\frac{SST-SSE}{SST}$$ (4)

where *SST* stands for the sum of squares for total, and *SSE* stands for the sum of the squares for error.

### Visualizing and statistical analyzing

The results were visualized using the Microsoft Excel software (2019), *ggvis* [74], and *ggplot2* [75] packages in the R software version 3.6.1. All statistical computational procedures were also conducted using *MASS* [76] package in R software.

## Results

### Pearson correlation analyses and individual ML evaluations

Based on the field data analyses, the average yield of 250 soybean genotypes was estimated to be between 2.58 to 5.71 ton ha^-1^ with a mean and standard deviation of 4.22 and 0.57 ton ha^-1^, respectively.

**Fig 3:**
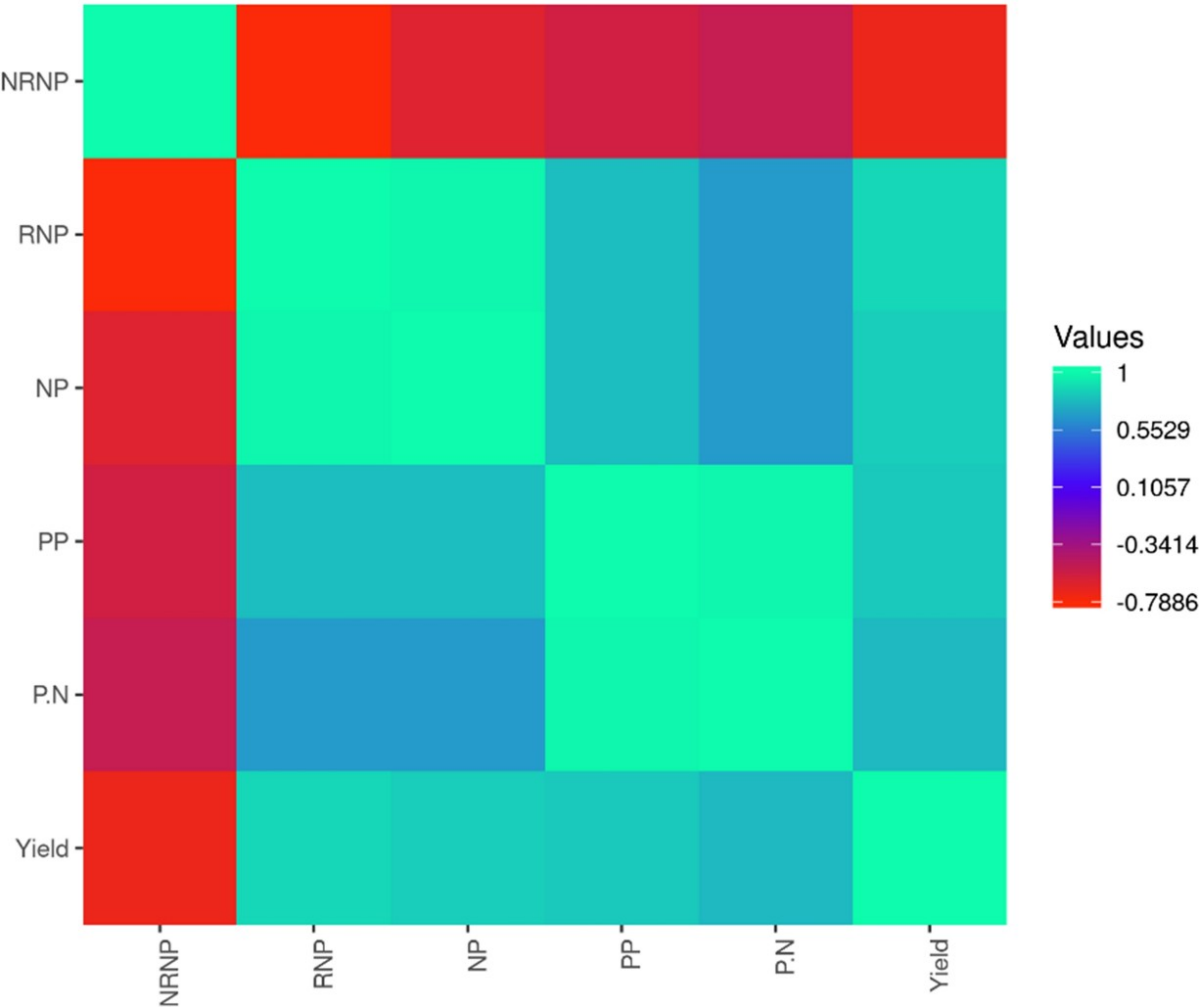
Pearson correlation analysis of soybean yield component traits. The Number of Nodes per plant (NP), the Number of Non-Reproductive Nodes per Plant (NRNP), the Number of Reproductive Nodes per Plant (RNP), and the Number of Pods per Plant (PP), the ratio of number of Pods to number of Nodes per plant (P.N).

The potential benefits of using each soybean yield component for predicting the soybean seed yield was quantified using the Pearson coefficients of correlation among all the measured traits. Based on the correlation coefficients, all the yield components, except NRNP, were positively correlated with soybean seed yield. The linear correlation between soybean seed yield and PP (*r* = 0.71) was found to be the strongest followed by its correlation with NP (*r* = 0.68), RNP (*r* = 0.67), and P/N (*r* = 0.64). The negative correlation between soybean seed yield and NRNP was estimated as *r* = -0.29 (Fig 3).

**Fig 4:**
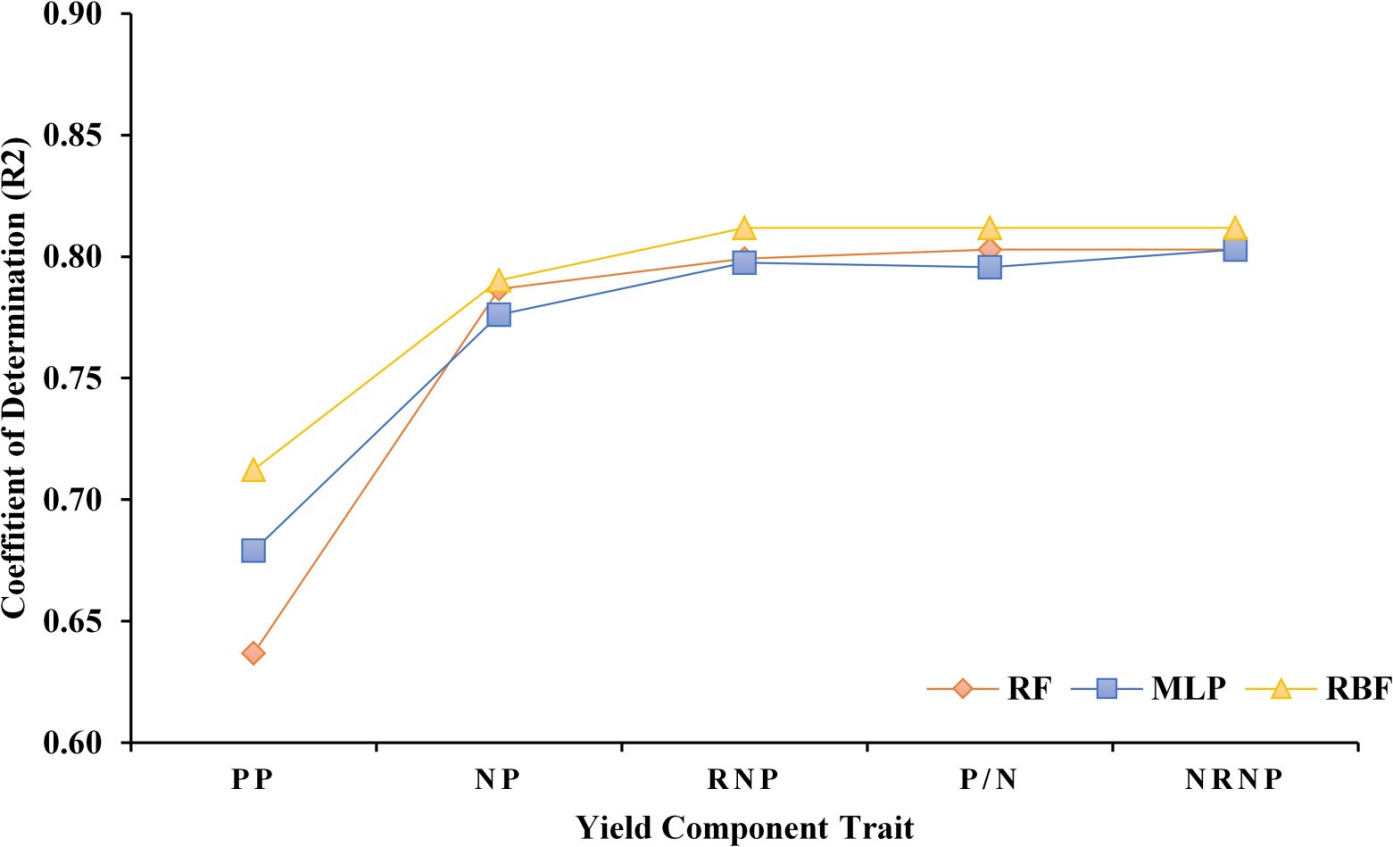
Model training accuracy for Random Forest (RF), Multilayer Perceptron (MLP), and Radial Basis Function (RBF) algorithms by adding variables based on the correlation results. The Number of Nodes per plant (NP), the Number of Non-Reproductive Nodes per Plant (NRNP), the Number of Reproductive Nodes per Plant (RNP), and the Number of Pods per Plant (PP), the ratio of number of Pods to number of Nodes per plant (P/N).

Based on the results of correlation analyses, the top correlated variables were iteratively added to the algorithms and updated the algorithm training performances until all variables were included. The R^2^ values of each model were then calculated (Fig 4). Among all the tested ML algorithms, the R^2^ reached its maximum value of 0.81 in RBF taking into account PP, NP, and RNP. No changes in R^2^ value were detected after adding P/N and NRNP in the RBF algorithm. The maximum R^2^ value of 0.80 was obtained for both RF and MLP when all the yield components are added into the algorithms (Fig 4).

### Model performance and evaluation

**Fig 5:**
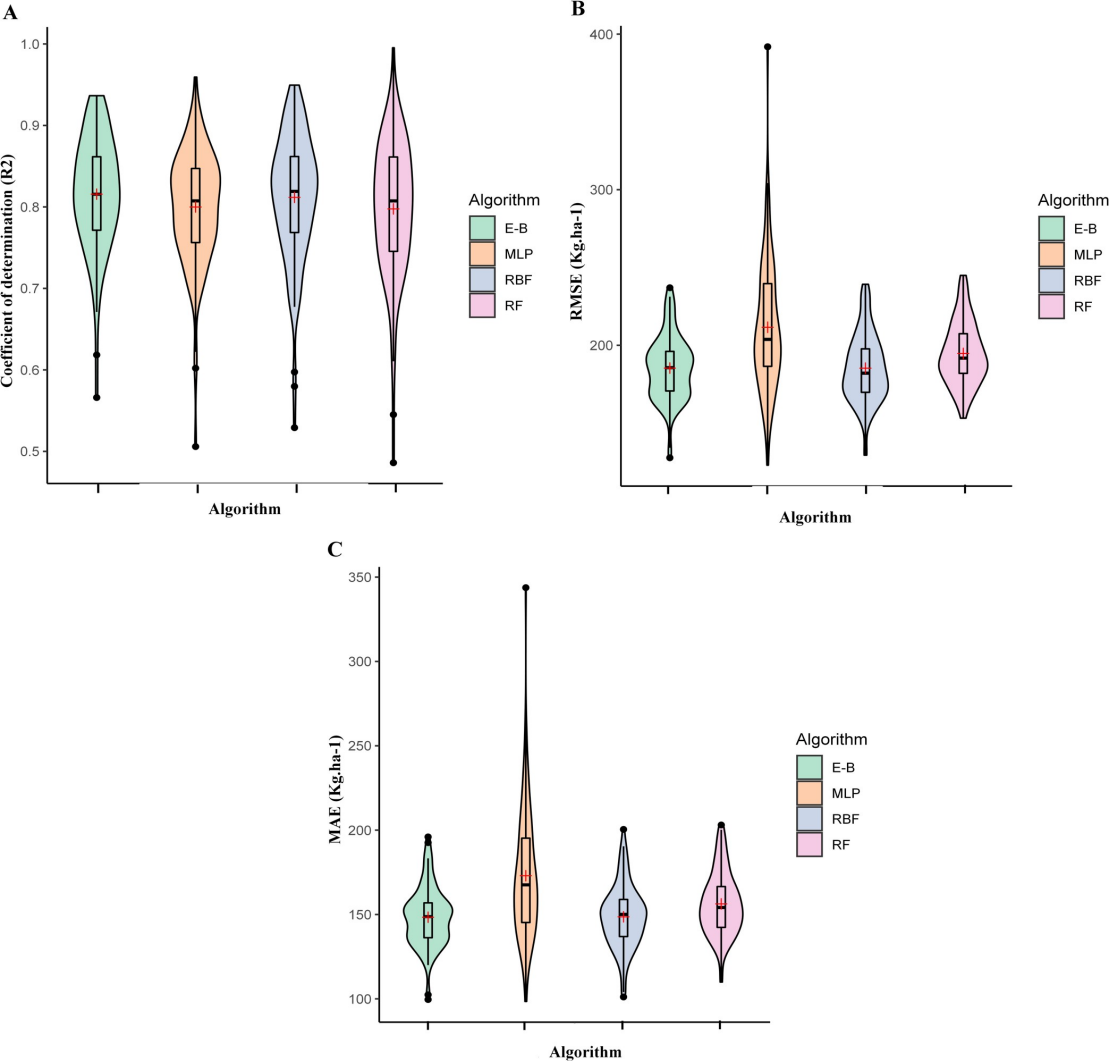
(A) coefficient of determination (R^2^), (B) the Root Mean Square Error (RMSE) and (C) the Mean Absolute Errors (MAE) performance of Random Forest (RF), Multilayer Perceptron (MLP), and Radial Basis Function (RBF) algorithms, and the Ensemble-Bagging (E-B) strategy for soybean yield prediction using yield component traits. The mean performance is indicated with an + sign in each Figure.

The full analysis of ML algorithms are presented in the S1 Table. Among the three tested ML algorithms, RBF had the highest value for R^2^ (0.81) and the lowest values for MAE (148.61 kg.ha^-1^) and RMSE (185.31 kg.ha^-1^) (Fig 5A–5C). The R^2^ values for MLP and RF were the same (0.80); however, they had different values for MAE and RMSE. In comparison with the MLP algorithm, RF had the lower MAE and RMSE with 156.28 kg.ha^-1^ and 194.75 kg.ha^-1^, respectively (Fig 5A–5C). MLP had the highest MAE (172.98 kg.ha^-1^), and RMSE (211.57 kg.ha^-1^) among the ML algorithms. In addition to individual evaluations of the three ML algorithms, an ensemble learning was also developed, which outperformed all the individual machine learning algorithms, attaining an R^2^ value of 0.82. The E-B method had the acceptable RMSE and MAE with a value of 184.35 kg.ha^-1^ and 148.37 kg.ha^-1^, respectively (Fig 5A–5C). In general, the R^2^ value of E-B increased by 0.1, while the values of MAE and RMAS decreased by 0.24 kg.ha^-1^ and 0.96 kg.ha^-1^, respectively, in comparison with RBF as the most accurate individual ML algorithm identified in this study.

### Optimization of the soybean seed yield using E-B-GA

The aim of the current study, not only was to predict soybean seed yield using yield components, but also to estimate the optimum values of these traits, i.e., NP, PP, RNP, NRNP, and P/N, to maximize the final yield production in a given genotype. The results of the optimization process using GA, as the single objective evolutionary optimization algorithm, are presented in Table 1. Theoretically, the highest soybean seed yield production of 5.64 ton ha^-1^ can be achieved in an ideotype soybean genotype in which the values of NP, NRNP, RNP, PP, and P/N are 17.32, 3.07, 14.25, 48.98, and 2.83, respectively.

**Table 1:** Optimizing the number of nodes per plant (NP), the number of non-reproductive nodes per plant (NRNP), the number of reproductive nodes per plant (RNP), and the number of pods per plant (PP), the ratio of number of pods to number of nodes per plant (P/N) according to the E-B-GA for maximizing soybean seed yield.

Input variables					Predicted Yield (ton ha^-1^)
NP	NRNP	RNP	PP	P/N	
17.32	3.07	14.25	48.98	2.83	5.64

## Discussion

One of the objectives of this study was to investigate the potential use of soybean yield components such as NP, PP, RNP, NRNP, and P/N for predicting the final seed yield production. Many studies reported the reliance of the final yield production on the performance of several yield-related traits [15, 77–80]. In soybean, PP and NP are considered as major players in determining the final seed yield [15, 81, 82]. In the current study, PP showed the highest linear correlation with the total seed yield. The direct impact of the number of pods per plant on the final soybean yield is also reported by Bastidas, Setiyono (83)]. Among all the tested yield component traits in this study, NP showed the second-highest linear correlation with total seed yield and showed a positive correlation with PP. Many studies reported that the variations in the number of nodes per plant is usually accounted for the changes in the number of pods per plant [81–84]. A negative correlation between the total soybean seed yield and NRNP was found in this study. It is in agreement with previous studies claimed that increasing the number of non-reproductive nodes decreases the reproductive potential of soybean seed yield [81, 85]. The results of linear correlation analyses in this study illustrated the importance of individual yield component traits in determining the total soybean seed yield.

**Fig 6:**
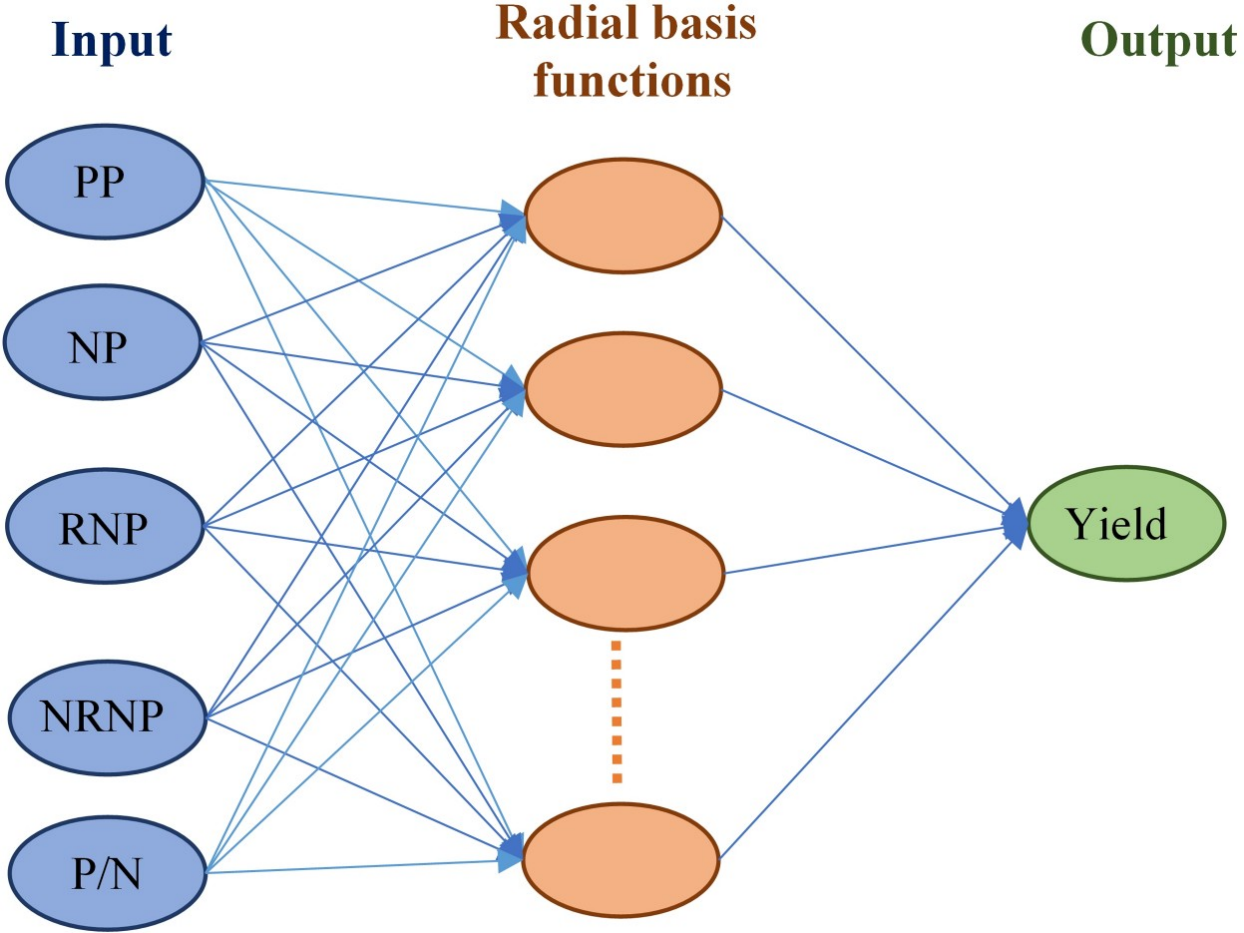
The schematic view of the Radial Basis Function (RBF) algorithm.

Conventional statistical methods such as ANOVA and simple regression methods are typically recommended for small datasets with limited dimensions [86, 87]. However, soybean yield is a complex trait under controlled by different continuous variables called yield component traits. Therefore, more sophisticated methods are required for analyzing large data sets with high dimensions [88]. The successful uses of ML algorithms for analyzing big data with multi-collinearity among the variables have recently been reported in many plant species such as soybean [30], alfalfa [48], chrysanthemum [89], wheat [90], and lime [91]. The prediction performance of a given machine learning algorithm refers to the power of the model in predicting the values of a dependent variable when non-representative samples, or samples from a different population, are used as the test population [92]. The prediction performance of an ML algorithm is estimated using its R^2^, RMSE, and MAE values [92–94]. In this study, the three common ML algorithms, RBF, MLP, and RF, were used to predict the soybean seed yield using its components and their prediction performance were estimated. RBF was found to be the most accurate ML algorithm for predicting the soybean seed yield from its component traits. In general, yield components in soybean are traits with low heritability that are influenced by environmental factors. The environmental factors can bring some levels of instability/noise in the results of all the ML analyses [95]. However, the structure of RBF (Fig 6) gives some level of robustness against the adversarial noises, compared to other tested ML algorithms [96, 97]. The specific structure of RBF is the use of the transfer function of input variables to hidden layer name radial basis function [64, 98]. This function plays an important role in reducing noises of input variables resulted in more accurate prediction performance [99].

Although RF and MLP had the same R^2^ values, the MAE and RMSE values were lower in RF. MLP, as one of the neural network algorithms, is highly susceptible to possible instabilities/noises caused by non-heritable factors. The MAE and RMSE values of this algorithm were the highest among all the tested ML algorithms that may indicate the sensitivity of the algorithm to noises. As a result, using this algorithm may not recommend for analyzing phenotypic data that are largely affected by environmental factors. RF with the R^2^, MAE, and RMSE values of 0.80, 156.28 kg.ha^-1^, and 194.75 kg.ha^-1^, respectively, was selected as the second-best ML algorithm for predicting soybean seed yield in this study. Although the difference between RF and RBF in terms of R^2^ values was not statistically significant (data are not shown), they were statistically different for their MAE and RMSE values. The performance of the RF algorithm relies on processing large dimensional data based on generalized error estimation [100, 101]. Also, there is no assumption requirement for RF about the distribution of data [102], and this algorithm can isolate outliers in a small region of the variable space resulted in acceptable performance against nonlinear environmental effects [102, 103].

In addition to individual comparison of the three tested ML algorithms, we developed a bagging ensemble model by combining the results of RBF, MLP, and RF in this study. Since the RBF had the highest performance in predicting the soybean seed yield, this algorithm was chosen as the metaClassifier for developing the E-B algorithm. Using the E-B model, a slight improvement was obtained in predicting the total soybean seed yield from its component traits. Diversity and sufficiency are two of the most important principles in selecting base learners for an ensemble model [67]. It means that the dependency among the used ML algorithms in the ensemble model should be minimized, while each based learner should have an acceptable predicting capability as well [104, 105]. Therefore, we selected different ML algorithms as the base learners for the E-B with different training data mechanisms. Also, the performance of the E-B was optimized by implementing RBF as the metaClassifier for this model. The effectiveness of using ensemble models was reported in different plant species such as chrysanthemum [106], sorghum [107], wheat [108], alfalfa [67], and brassicas [109]. This study demonstrates the benefit of using the RBF-based E-B approach to improve the soybean yield prediction accuracy using yield components.

Selecting high-yielding lines has always been one of the major goals in plant breeding programs that can be performed using either direct or indirect selection approaches [110]. Selecting superior genotypes based on the yield component traits can be considered as an indirect method. An analytical breeding strategy is an alternate breeding approach that is focused on the improvement of components of complex traits such as plant growth and development rates or yield components [111] rather than the traits *per se*. This strategy can improve genetic yield potential in varieties by setting up selection criteria on yield components and making the selection more efficient [112]. In order to move toward analytical breeding, it would be important to have the optimized level of each yield component traits in target populations. Genetic algorithm is commonly used in finding optimized solutions by searching problems through biological parameters such as selection, crossover, and mutation [53, 113]. After selecting E-B as the combined algorithm with the highest prediction ability in this study, GA was linked into this algorithm to estimate the optimum values of the yield component traits (Table 1). The successfulness of using the GA algorithm for estimating optimized solutions was reported previously in plant tissue culture [89], plant physiology [114], and remote sensing [115].

## Conclusion

Efficient breeding approaches for improving the genetic potential of complex traits such as yield in soybean can be developed based upon secondary traits that govern the final performance of the complex traits. Thus, in order to increase the genetic yield potential in soybean, breeders can select soybean genotypes with improved yield component traits. However, measuring yield components in a large soybean breeding program is time-consuming and labor-intensive, which limit the implication of this approach in cultivar development programs. The main objective of this study was to evaluate the potential use of yield component traits for estimating final seed yield in soybean using different ML and E-B algorithms, which in turn can be used by breeders for selecting parental lines and designing promising crosses for developing cultivars with improved genetic yield potential. The results of the current study showed that RBF is a reliable solo ML algorithm for predicting the soybean seed yield from its component traits. However, an E-B algorithm that was built by combining the three ML and using RBF as its metaClassifier outperformed all individual ML algorithms and, therefore, it is recommended for predicting the soybean seed yield exploiting yield component traits. In the current study for the first time, we coupled E-B algorithm with GA in order to estimate optimum values of yield component traits in a theoretical genotype in which the yield is maximized using the real field data. The results seem to be promising but are recommended to be evaluated in new and independent breeding populations before using in cultivar development programs for selecting high-yielding potential genotypes. This information can be also used, in combined with molecular marker technology, for developing genomic-based toolkits that can be used for selecting genotypes with improved genetic yield potential at early generations.

## Supporting information

S1 Table Analysis performance of Random Forest (RF), Multilayer Perceptron (MLP), and Radial Basis Function (RBF) algorithms, and the Ensemble-Bagging (E-B) strategy for soybean yield prediction using yield component traits. (DOCX)
