# Supplementary material for: Application of machine learning and genetic optimization algorithms for modeling and optimizing soybean yield using its component traits
Source: PLoS One. 2021 Apr 30;16(4):e0250665. doi: 10.1371/journal.pone.0250665 (PMC8087002; doi:10.1371/journal.pone.0250665)
Supplement: S1 Table — (DOCX) [file pone.0250665.s001.docx]

**Application of Machine Learning and Genetic Optimization Algorithms for Modeling and Optimizing Soybean Yield Using its Component Traits**

**Mohsen Yoosefzadeh-Najafabadi,^1^ Dan Tulpan,^2^ Milad Eskandari^1*^**

^1.^ Department of Plant Agriculture, University of Guelph, Guelph, ON N1G 2W1, Canada

^2.^ Department of Animal Biosciences, University of Guelph, Guelph, ON N1G 2W1, Canada

**^*^**Corresponding author.

Email: [meskanda@uoguelph.ca](file:///C:\Users\dtulpan\UofG%20Work\Papers\2019\Mohsen%20Y\meskanda@uoguelph.ca)

| **S1 Table. Analysis performance of Random Forest (RF), Multilayer Perceptron (MLP), and Radial Basis Function (RBF) algorithms, and the Ensemble-Bagging (E-B) strategy for soybean yield prediction using yield component traits.** | | | |
| --- | --- | --- | --- |
| **Algorithm** | **MAE (Kg.ha^-1^)** | **RMSE (Kg.ha^-1^)** | **Coefficient of determination (R^2^)** |
| RF | 178.7628 | 221.3154 | 0.766184 |
| RF | 149.9257 | 190.1973 | 0.824873 |
| RF | 195.2948 | 244.9381 | 0.783472 |
| RF | 158.4371 | 186.9633 | 0.545133 |
| RF | 162.9635 | 197.5527 | 0.820895 |
| RF | 141.4723 | 177.8491 | 0.892779 |
| RF | 136.1359 | 175.7995 | 0.87027 |
| RF | 190.0622 | 232.3531 | 0.692505 |
| RF | 138.5809 | 176.5637 | 0.804284 |
| RF | 159.3969 | 182.2693 | 0.812292 |
| RF | 148.2689 | 182.6683 | 0.900656 |
| RF | 167.9388 | 207.3602 | 0.800588 |
| RF | 154.2294 | 187.3587 | 0.714045 |
| RF | 168.8059 | 206.1508 | 0.836931 |
| RF | 143.3354 | 185.6513 | 0.825753 |
| RF | 164.4261 | 193.7816 | 0.726271 |
| RF | 145.82 | 168.8407 | 0.930298 |
| RF | 148.9438 | 205.5292 | 0.75625 |
| RF | 157.872 | 203.142 | 0.736331 |
| RF | 146.8995 | 196.8535 | 0.724496 |
| RF | 177.6075 | 222.6089 | 0.87656 |
| RF | 143.2936 | 185.0717 | 0.747739 |
| RF | 130.328 | 159.4461 | 0.889543 |
| RF | 133.714 | 171.697 | 0.890703 |
| RF | 188.8504 | 218.2928 | 0.708521 |
| RF | 161.8679 | 196.3354 | 0.825907 |
| RF | 153.3627 | 191.2781 | 0.772181 |
| RF | 160.157 | 205.1084 | 0.857359 |
| RF | 200.2354 | 236.3703 | 0.61441 |
| RF | 127.769 | 169.0819 | 0.995297 |
| RF | 182.285 | 220.6546 | 0.856008 |
| RF | 149.9078 | 177.8497 | 0.749269 |
| RF | 148.4835 | 184.1446 | 0.738688 |
| RF | 142.302 | 187.7328 | 0.871173 |
| RF | 163.3174 | 214.4299 | 0.909112 |
| RF | 196.0316 | 242.842 | 0.709007 |
| RF | 150.1975 | 188.1803 | 0.807753 |
| RF | 150.36 | 186.1467 | 0.867454 |
| RF | 142.3568 | 173.0309 | 0.768005 |
| RF | 140.9918 | 177.5659 | 0.818197 |
| RF | 148.6688 | 186.3253 | 0.786482 |
| RF | 148.4959 | 186.2586 | 0.863586 |
| RF | 138.4413 | 164.3384 | 0.81034 |
| RF | 170.5698 | 214.3142 | 0.829237 |
| RF | 165.9738 | 204.0655 | 0.76936 |
| RF | 175.2973 | 212.9283 | 0.732842 |
| RF | 154.5922 | 199.6483 | 0.779811 |
| RF | 164.71 | 213.779 | 0.868679 |
| RF | 141.0413 | 191.7986 | 0.829673 |
| RF | 163.0976 | 191.7426 | 0.738629 |
| RF | 148.0027 | 178.9045 | 0.836692 |
| RF | 181.4252 | 223.3686 | 0.784269 |
| RF | 152.5579 | 189.5512 | 0.82343 |
| RF | 170.461 | 207.7199 | 0.734849 |
| RF | 141.7312 | 176.5751 | 0.735335 |
| RF | 157.3465 | 200.8962 | 0.869518 |
| RF | 145.9078 | 187.7716 | 0.875962 |
| RF | 181.4088 | 213.8097 | 0.688733 |
| RF | 136.9581 | 170.6061 | 0.817263 |
| RF | 128.4578 | 167.8907 | 0.833361 |
| RF | 134.8502 | 184.0278 | 0.898587 |
| RF | 153.402 | 200.3877 | 0.797845 |
| RF | 141.3659 | 173.3029 | 0.876889 |
| RF | 135.4279 | 167.1626 | 0.757907 |
| RF | 158.5733 | 183.4167 | 0.813396 |
| RF | 182.4223 | 227.7454 | 0.929062 |
| RF | 156.8169 | 202.0108 | 0.752552 |
| RF | 189.7572 | 230.0217 | 0.610582 |
| RF | 147.8972 | 184.1388 | 0.844133 |
| RF | 150.5905 | 210.643 | 0.790229 |
| RF | 170.2709 | 212.5736 | 0.735781 |
| RF | 166.4571 | 199.5631 | 0.883256 |
| RF | 139.4238 | 178.0832 | 0.800048 |
| RF | 157.0009 | 196.2237 | 0.860542 |
| RF | 135.3621 | 169.3442 | 0.844932 |
| RF | 178.0886 | 222.583 | 0.77273 |
| RF | 144.9649 | 185.4615 | 0.691444 |
| RF | 174.6506 | 210.4431 | 0.764172 |
| RF | 163.1946 | 200.6054 | 0.807037 |
| RF | 115.3843 | 153.158 | 0.890578 |
| RF | 168.763 | 193.9537 | 0.838 |
| RF | 159.3417 | 214.9731 | 0.646471 |
| RF | 160.0949 | 202.2082 | 0.783883 |
| RF | 181.7538 | 218.347 | 0.737263 |
| RF | 151.244 | 185.6695 | 0.820413 |
| RF | 166.8557 | 192.8271 | 0.893896 |
| RF | 132.159 | 169.5062 | 0.70914 |
| RF | 131.7293 | 160.3757 | 0.893691 |
| RF | 133.2249 | 174.1565 | 0.762251 |
| RF | 193.9929 | 236.8308 | 0.816991 |
| RF | 165.0995 | 197.0843 | 0.769782 |
| RF | 140.4964 | 183.8125 | 0.936378 |
| RF | 154.0391 | 182.249 | 0.869982 |
| RF | 109.9387 | 158.5995 | 0.917128 |
| RF | 163.8293 | 201.346 | 0.657122 |
| RF | 139.5893 | 181.1232 | 0.857129 |
| RF | 154.5968 | 191.6562 | 0.713304 |
| RF | 155.9699 | 193.7374 | 0.783702 |
| RF | 203.071 | 239.3553 | 0.486035 |
| RF | 150.6367 | 193.4698 | 0.698959 |
| MLP | 172.7595 | 217.5933 | 0.780432 |
| MLP | 200.6261 | 252.9527 | 0.801682 |
| MLP | 169.3347 | 218.3947 | 0.857704 |
| MLP | 166.7933 | 203.4841 | 0.602116 |
| MLP | 144.2937 | 190.6019 | 0.848869 |
| MLP | 143.857 | 170.3943 | 0.753572 |
| MLP | 126.8736 | 164.798 | 0.705991 |
| MLP | 231.2917 | 284.9384 | 0.828591 |
| MLP | 226.1779 | 268.8094 | 0.831738 |
| MLP | 231.1072 | 269.5651 | 0.791386 |
| MLP | 201.0398 | 246.1417 | 0.881213 |
| MLP | 167.2376 | 197.22 | 0.866975 |
| MLP | 158.2354 | 201.3958 | 0.737549 |
| MLP | 190.92 | 218.587 | 0.86469 |
| MLP | 148.1788 | 186.9506 | 0.842144 |
| MLP | 146.7581 | 191.6821 | 0.756363 |
| MLP | 137.9938 | 157.613 | 0.755116 |
| MLP | 208.8802 | 241.2391 | 0.786219 |
| MLP | 159.0894 | 209.0648 | 0.815781 |
| MLP | 145.0941 | 203.2909 | 0.744231 |
| MLP | 187.699 | 229.2773 | 0.86999 |
| MLP | 216.459 | 261.1971 | 0.756014 |
| MLP | 135.9898 | 177.9439 | 0.864208 |
| MLP | 235.367 | 262.5487 | 0.804906 |
| MLP | 164.5036 | 187.6228 | 0.774705 |
| MLP | 169.4086 | 217.1101 | 0.850425 |
| MLP | 135.6038 | 160.3304 | 0.853235 |
| MLP | 174.78 | 196.1875 | 0.716394 |
| MLP | 161.6382 | 196.0674 | 0.706674 |
| MLP | 98.44332 | 122.9621 | 0.842102 |
| MLP | 186.9764 | 245.0146 | 0.822978 |
| MLP | 109.0222 | 137.5278 | 0.770514 |
| MLP | 166.0958 | 207.0647 | 0.796704 |
| MLP | 202.1948 | 229.1342 | 0.828122 |
| MLP | 201.4795 | 242.5588 | 0.786087 |
| MLP | 235.1983 | 288.2606 | 0.73038 |
| MLP | 153.1835 | 184.1012 | 0.82238 |
| MLP | 155.4043 | 194.6755 | 0.769831 |
| MLP | 261.0962 | 304.2983 | 0.783093 |
| MLP | 152.1932 | 183.7509 | 0.828472 |
| MLP | 161.1597 | 212.5492 | 0.778382 |
| MLP | 118.8771 | 164.4517 | 0.768988 |
| MLP | 132.8556 | 157.3079 | 0.77531 |
| MLP | 190.1359 | 233.156 | 0.862115 |
| MLP | 167.8941 | 207.3835 | 0.783019 |
| MLP | 168.872 | 205.7792 | 0.74932 |
| MLP | 151.1987 | 195.7471 | 0.780283 |
| MLP | 170.4522 | 198.8213 | 0.914687 |
| MLP | 146.5039 | 196.3535 | 0.828794 |
| MLP | 130.429 | 157.1896 | 0.831685 |
| MLP | 148.6512 | 188.2507 | 0.825356 |
| MLP | 173.4913 | 212.3744 | 0.806248 |
| MLP | 168.5609 | 211.869 | 0.793968 |
| MLP | 166.3879 | 196.8307 | 0.774842 |
| MLP | 145.2993 | 188.9879 | 0.716266 |
| MLP | 149.7417 | 188.7923 | 0.889262 |
| MLP | 132.4694 | 158.6575 | 0.902521 |
| MLP | 169.8295 | 205.7533 | 0.7742 |
| MLP | 195.2361 | 240.853 | 0.809339 |
| MLP | 165.2936 | 188.3967 | 0.720568 |
| MLP | 137.1391 | 179.4708 | 0.711557 |
| MLP | 343.7492 | 391.8631 | 0.813609 |
| MLP | 133.5503 | 162.2748 | 0.866505 |
| MLP | 131.7657 | 164.7783 | 0.748885 |
| MLP | 136.6483 | 178.058 | 0.847794 |
| MLP | 177.7942 | 216.2273 | 0.755567 |
| MLP | 144.0417 | 175.1276 | 0.844891 |
| MLP | 195.2948 | 251.2979 | 0.622138 |
| MLP | 143.8909 | 180.552 | 0.887983 |
| MLP | 156.2171 | 211.643 | 0.807022 |
| MLP | 197.7098 | 233.4665 | 0.766644 |
| MLP | 168.9159 | 202.061 | 0.905567 |
| MLP | 202.618 | 246.1472 | 0.824288 |
| MLP | 209.1927 | 254.7927 | 0.846966 |
| MLP | 171.9806 | 214.3512 | 0.841538 |
| MLP | 156.5163 | 195.6712 | 0.827064 |
| MLP | 189.4516 | 219.3236 | 0.68501 |
| MLP | 202.7257 | 239.2442 | 0.807838 |
| MLP | 222.0311 | 249.4734 | 0.86223 |
| MLP | 164.8277 | 187.0467 | 0.892593 |
| MLP | 163.8334 | 198.9956 | 0.856719 |
| MLP | 142.352 | 190.0295 | 0.739342 |
| MLP | 185.9694 | 234.6922 | 0.834398 |
| MLP | 207.9813 | 253.4011 | 0.761064 |
| MLP | 242.326 | 292.8608 | 0.854342 |
| MLP | 142.567 | 174.978 | 0.707284 |
| MLP | 175.0444 | 203.6102 | 0.753441 |
| MLP | 154.5905 | 180.7022 | 0.88873 |
| MLP | 175.7215 | 230.6778 | 0.810041 |
| MLP | 181.9961 | 221.9387 | 0.834332 |
| MLP | 204.4311 | 253.3968 | 0.780615 |
| MLP | 121.9059 | 150.4393 | 0.959347 |
| MLP | 176.2471 | 203.9698 | 0.838498 |
| MLP | 220.3621 | 245.4573 | 0.918385 |
| MLP | 143.5251 | 185.0442 | 0.704066 |
| MLP | 193.3929 | 247.4376 | 0.858957 |
| MLP | 133.1788 | 169.0064 | 0.751028 |
| MLP | 141.1103 | 169.8037 | 0.83664 |
| MLP | 215.6617 | 270.1576 | 0.505873 |
| MLP | 257.6033 | 296.671 | 0.715667 |
| RBF | 183.6138 | 233.221 | 0.742965 |
| RBF | 154.2808 | 193.7745 | 0.820005 |
| RBF | 143.5776 | 212.9047 | 0.830351 |
| RBF | 151.1534 | 180.7979 | 0.579802 |
| RBF | 142.3292 | 173.993 | 0.855718 |
| RBF | 143.5067 | 172.3785 | 0.900257 |
| RBF | 129.8851 | 170.7283 | 0.879767 |
| RBF | 158.327 | 188.5379 | 0.80311 |
| RBF | 137.4389 | 169.0885 | 0.818078 |
| RBF | 142.8674 | 173.7702 | 0.834642 |
| RBF | 155.428 | 208.4045 | 0.859551 |
| RBF | 127.8367 | 167.6985 | 0.868803 |
| RBF | 155.2717 | 187.98 | 0.72971 |
| RBF | 159.2594 | 196.2965 | 0.851342 |
| RBF | 145.7332 | 186.4238 | 0.823136 |
| RBF | 140.1722 | 181.7567 | 0.762889 |
| RBF | 136.9643 | 158.6364 | 0.935984 |
| RBF | 161.9318 | 197.444 | 0.771353 |
| RBF | 150.1286 | 189.948 | 0.789082 |
| RBF | 157.2294 | 215.6805 | 0.706224 |
| RBF | 163.7579 | 213.0716 | 0.881643 |
| RBF | 136.7781 | 173.1275 | 0.781717 |
| RBF | 129.7098 | 171.1569 | 0.869125 |
| RBF | 138.8365 | 175.307 | 0.875032 |
| RBF | 151.6126 | 178.6148 | 0.795283 |
| RBF | 158.8418 | 199.1415 | 0.843254 |
| RBF | 126.6348 | 157.6121 | 0.840598 |
| RBF | 142.4766 | 187.3825 | 0.710544 |
| RBF | 200.4364 | 229.3553 | 0.677522 |
| RBF | 103.9961 | 129.3797 | 0.931323 |
| RBF | 141.2857 | 169.9702 | 0.921905 |
| RBF | 140.7124 | 168.8831 | 0.782043 |
| RBF | 137.1646 | 168.7338 | 0.782994 |
| RBF | 133.134 | 164.1681 | 0.922924 |
| RBF | 178.4739 | 230.8923 | 0.768754 |
| RBF | 190.426 | 233.797 | 0.74503 |
| RBF | 133.7204 | 171.3819 | 0.808639 |
| RBF | 154.9224 | 196.7903 | 0.871257 |
| RBF | 150.331 | 172.045 | 0.775318 |
| RBF | 135.5637 | 166.4849 | 0.843777 |
| RBF | 142.8767 | 181.9715 | 0.795573 |
| RBF | 127.5752 | 161.77 | 0.895073 |
| RBF | 136.9867 | 169.1653 | 0.743812 |
| RBF | 168.7578 | 204.0289 | 0.854152 |
| RBF | 162.0003 | 197.4827 | 0.784401 |
| RBF | 176.6895 | 222.8623 | 0.717401 |
| RBF | 159.6183 | 206.0398 | 0.759635 |
| RBF | 151.3934 | 177.5788 | 0.911078 |
| RBF | 154.743 | 200.9931 | 0.818175 |
| RBF | 141.6372 | 172.916 | 0.801914 |
| RBF | 153.4421 | 198.3745 | 0.813678 |
| RBF | 162.2073 | 196.0599 | 0.836618 |
| RBF | 161.3724 | 199.8997 | 0.812045 |
| RBF | 170.6379 | 199.1354 | 0.762521 |
| RBF | 153.5326 | 187.0526 | 0.690971 |
| RBF | 144.5773 | 181.9399 | 0.892574 |
| RBF | 129.3503 | 166.7908 | 0.908665 |
| RBF | 150.9481 | 183.4863 | 0.77557 |
| RBF | 143.3755 | 182.2115 | 0.812386 |
| RBF | 101.1158 | 132.3554 | 0.909606 |
| RBF | 116.2528 | 159.9406 | 0.929833 |
| RBF | 150.3993 | 196.5136 | 0.812119 |
| RBF | 142.125 | 168.2098 | 0.870993 |
| RBF | 133.3209 | 165.6612 | 0.743889 |
| RBF | 148.696 | 170.7859 | 0.844676 |
| RBF | 177.7905 | 215.8261 | 0.755023 |
| RBF | 150.9376 | 181.0354 | 0.808973 |
| RBF | 181.3793 | 239.2106 | 0.597383 |
| RBF | 125.9981 | 158.3065 | 0.885233 |
| RBF | 159.148 | 207.9001 | 0.79668 |
| RBF | 168.702 | 209.1337 | 0.734244 |
| RBF | 150.6802 | 180.0562 | 0.913315 |
| RBF | 130.665 | 168.7718 | 0.834703 |
| RBF | 176.3399 | 211.3994 | 0.845549 |
| RBF | 130.1258 | 164.8486 | 0.855814 |
| RBF | 158.3807 | 194.6542 | 0.82766 |
| RBF | 149.738 | 187.2562 | 0.688145 |
| RBF | 155.8235 | 196.5279 | 0.806485 |
| RBF | 135.5634 | 172.8242 | 0.850478 |
| RBF | 118.5061 | 150.6716 | 0.892534 |
| RBF | 163.792 | 192.0888 | 0.85329 |
| RBF | 162.8707 | 206.0287 | 0.677389 |
| RBF | 153.2241 | 183.6246 | 0.827782 |
| RBF | 169.817 | 207.3453 | 0.768112 |
| RBF | 148.8906 | 178.6115 | 0.838454 |
| RBF | 151.5278 | 177.2087 | 0.8991 |
| RBF | 117.3948 | 152.5232 | 0.75611 |
| RBF | 143.7546 | 174.0319 | 0.882123 |
| RBF | 107.5289 | 157.072 | 0.81195 |
| RBF | 189.9371 | 224.5631 | 0.831525 |
| RBF | 150.3458 | 190.7095 | 0.783228 |
| RBF | 124.2933 | 153.913 | 0.949476 |
| RBF | 168.0047 | 193.4269 | 0.85032 |
| RBF | 123.3812 | 159.9852 | 0.923831 |
| RBF | 145.5034 | 182.8422 | 0.692957 |
| RBF | 153.3721 | 185.3318 | 0.851531 |
| RBF | 150.0027 | 190.8469 | 0.69351 |
| RBF | 134.2136 | 167.6947 | 0.835008 |
| RBF | 187.8876 | 233.8888 | 0.529072 |
| RBF | 136.4447 | 181.0933 | 0.734279 |
| E-B | 183.2795 | 226.4269 | 0.760125 |
| E-B | 158.7388 | 197.9632 | 0.811211 |
| E-B | 138.9816 | 195.5484 | 0.85706 |
| E-B | 157.427 | 184.4039 | 0.566074 |
| E-B | 153.1076 | 182.1446 | 0.842777 |
| E-B | 135.9 | 166.1241 | 0.909051 |
| E-B | 125.8501 | 166.0372 | 0.883882 |
| E-B | 153.9751 | 185.6232 | 0.806269 |
| E-B | 132.8204 | 162.7451 | 0.830713 |
| E-B | 152.0976 | 182.3546 | 0.809535 |
| E-B | 152.3521 | 197.8197 | 0.870155 |
| E-B | 132.6661 | 172.5216 | 0.861593 |
| E-B | 152.6118 | 179.918 | 0.734378 |
| E-B | 157.1289 | 191.2173 | 0.858173 |
| E-B | 151.6467 | 187.7465 | 0.821321 |
| E-B | 156.2297 | 189.5207 | 0.739623 |
| E-B | 134.4548 | 158.9776 | 0.93652 |
| E-B | 156.8858 | 189.8088 | 0.789772 |
| E-B | 151.1254 | 189.4081 | 0.793575 |
| E-B | 137.703 | 194.9087 | 0.727458 |
| E-B | 167.3076 | 221.0777 | 0.877 |
| E-B | 136.2339 | 170.8039 | 0.787356 |
| E-B | 128.6773 | 170.656 | 0.873827 |
| E-B | 138.0543 | 176.1757 | 0.874227 |
| E-B | 154.1638 | 183.4862 | 0.783918 |
| E-B | 154.8244 | 193.3933 | 0.844108 |
| E-B | 136.871 | 164.7964 | 0.832547 |
| E-B | 158.5593 | 200.0827 | 0.671116 |
| E-B | 192.5827 | 218.3656 | 0.707427 |
| E-B | 102.409 | 127.7135 | 0.934456 |
| E-B | 132.2051 | 170.6688 | 0.927864 |
| E-B | 140.9156 | 168.9344 | 0.777631 |
| E-B | 136.3336 | 165.9878 | 0.789646 |
| E-B | 133.3576 | 164.0796 | 0.922245 |
| E-B | 182.7726 | 231.2405 | 0.76968 |
| E-B | 195.9902 | 236.992 | 0.744964 |
| E-B | 137.6149 | 174.0405 | 0.810837 |
| E-B | 143.9593 | 189.4716 | 0.870914 |
| E-B | 148.7182 | 172.3549 | 0.771355 |
| E-B | 134.8865 | 168.0054 | 0.839248 |
| E-B | 150.7692 | 193.0609 | 0.771416 |
| E-B | 126.5058 | 159.9068 | 0.898655 |
| E-B | 129.4275 | 161.363 | 0.76353 |
| E-B | 168.6478 | 203.2126 | 0.855768 |
| E-B | 156.2701 | 198.9763 | 0.776954 |
| E-B | 173.3665 | 215.7703 | 0.732595 |
| E-B | 157.8172 | 200.3769 | 0.777714 |
| E-B | 152.7948 | 199.1369 | 0.900538 |
| E-B | 146.3063 | 194.6961 | 0.819759 |
| E-B | 135.8349 | 165.4279 | 0.814491 |
| E-B | 148.6061 | 193.467 | 0.811268 |
| E-B | 164.4626 | 209.4214 | 0.817501 |
| E-B | 166.5085 | 208.0148 | 0.800263 |
| E-B | 160.7459 | 193.2639 | 0.769775 |
| E-B | 152.2019 | 182.9039 | 0.707946 |
| E-B | 147.0838 | 185.9628 | 0.888372 |
| E-B | 125.6281 | 164.7244 | 0.914783 |
| E-B | 153.7599 | 186.1348 | 0.769928 |
| E-B | 146.1042 | 184.4388 | 0.808546 |
| E-B | 99.57982 | 134.1271 | 0.918512 |
| E-B | 123.7468 | 182.1538 | 0.911804 |
| E-B | 149.7901 | 193.9552 | 0.815593 |
| E-B | 140.4084 | 171.2181 | 0.860484 |
| E-B | 132.9433 | 163.8346 | 0.750176 |
| E-B | 144.8486 | 165.7402 | 0.857833 |
| E-B | 182.0624 | 223.981 | 0.737045 |
| E-B | 138.1152 | 166.9749 | 0.834598 |
| E-B | 182.5969 | 228.1908 | 0.618374 |
| E-B | 135.1193 | 164.8735 | 0.87525 |
| E-B | 155.9421 | 203.3862 | 0.800791 |
| E-B | 170.2542 | 205.8841 | 0.742724 |
| E-B | 145.8695 | 175.8282 | 0.913357 |
| E-B | 139.8621 | 179.9638 | 0.807157 |
| E-B | 170.7393 | 207.0985 | 0.850856 |
| E-B | 140.3855 | 177.5433 | 0.839968 |
| E-B | 146.8663 | 190.462 | 0.827966 |
| E-B | 152.8863 | 192.7258 | 0.685114 |
| E-B | 152.4733 | 194.3408 | 0.802668 |
| E-B | 131.9154 | 172.8414 | 0.858238 |
| E-B | 120.0153 | 152.5974 | 0.893512 |
| E-B | 168.0297 | 196.0895 | 0.847438 |
| Coefficient of determination (R^2^), the Root Mean Square Error (RMSE) and the Mean Absolute Errors (MAE) | | | |
